# Characterization of complete mitochondrial genome of seven-eleven crab *Carpilius maculatus* (Linnaeus, 1758)

**DOI:** 10.1080/23802359.2019.1679682

**Published:** 2019-10-21

**Authors:** Hongtao Liu, Mingqiu Yang, Yugui He

**Affiliations:** Hainan Provincial Key Laboratory of Tropical Maricultural Technologies, Hainan Academy of Ocean and Fisheries Sciences, Haikou, China

**Keywords:** *Carpilius maculatus*, mitochondrial genome, phylogenetic analysis

## Abstract

In this study, we first determined and characterised the complete mitochondrial genome of seven-eleven crab *Carpilius maculatus* from South China Sea. The *C. maculatus* mitogenome is 15,761 bp long, and consists of 22 tRNA genes, two rRNA genes, 13 protein-coding genes (PCGs), and one control region. The nucleotide composition of *C. maculatus* mitogenome is significantly biased (A, G, T, and C was 35.9%, 9.8%, 35.5%, and 18.9%, respectively) with A + T contents of 71.3%. Three PCGs used an unusual initiation codon, and four PCGs were terminated with an uncomplete or abnormal stop codon. Six microsatellites were identified in *C. maculatus* mitogenome sequences. Phylogenetic tree showed that *C. maculatus* formed monophyletic clade with other Heterotremata species.

*Carpilius maculatus*, common names seven-eleven crab or spotted reef crab, belongs to the family Carpiliidae. It is probably the most prominent representative of its genus, on its surface with 11 symmetrically disposed red blots (Raju et al. 2015). It is a reef crab, and found on coral and rocky reefs in Indo-West Pacific moving slowly along sandy bottoms (Zacharia et al. 2008). They feed nocturnally on marine snails. Although occasionally offered on the Southeast Asian markets as food, and there are reports that it is poisonous, but so far it could also by biochemical tests have not been confirmed (Holthuis 1968; Halstead and Cox 1973). It is considered that the crab possible by eating of poisonous snails will also be toxic, but even this is not confirmed. In addition, the first-stage zoaea of *C. maculatus* has also been studied (Clark et al. 2004).

The samples were collected from Huanqiu wharf of Wenchang, China (N19°33′51.12″, E110°49′27.98″), and stored in the marine crustacean specimen room (C20190616CM) in Qionghai research base of Hainan Academy of Ocean and Fisheries Sciences for reference, Muscle samples of *C. maculatus* were preserved in absolute ethanol for total DNA extraction.

The whole mitogenome of *C. maculatus* is 15,761 bp in size (GenBank Accession No. MN381805). The base content was 35.9% A, 9.8% G, 35.5% T, and 18.9% C. The 71.3% of (A + T) showed great preference to AT. It consists of 22 tRNA genes, two rRNA genes, 13 protein-coding genes (PCGs), and one control region (D-loop). Four PCGs (ND1, ND4, ND4L and ND5), eight tRNA genes and two rRNA genes were located on the light strand, the others were encoded by the heavy strand.

The 22 tRNA genes in mitogenome of *C. maculatus* vary in length from 63 bp to 74 bp. tRNA-Leu and tRNA-Ser both have two type copies respectively. The 12S rRNA is 843 bp and located between tRNA-Val and D-loop, and the 16S rRNA is 1342 bp, located between tRNA-Val and tRNA-Leu. Except ND1 using an unusual GTG as the start codon, the others use a normal initiation codon ATN. Simultaneously, most PCGs were terminated with a usual codon TAA or TAG in addition to COX2 and CYTB genes using an incomplete stop codon TT or T respectively. The control region is 758 bp, located between 12S rRNA and tRNA-Ile. Interestingly, we identified six microsatellites (SSRs) in *C. maculatus* mitogenome using MISA. A (A)_10_ is located in ND1 genes, a (TA)_6_ in ND4 genes, a (T)_10_ in 16S rRNA, a (A)_10_ and (AT)_7_ both in D-loop region, and a (T)_10_ is sited in non-coding sequences.

A maximum likelihood phylogenetic analysis with 36 crab mitogenomes placed *C. maculatus* among Heterotremata species with 1000 bootstrap replicates. The result (Figure 1) showed that Carpilioidea represented by *C. maculatus* formed monophyletic clade with other Heterotremata species, and further clarified the phylogenetic relationships of the superfamily in Heterotremata compared with the previous work (Wetzer et al. 2003; Lai et al. 2014).

**Figure 1.**
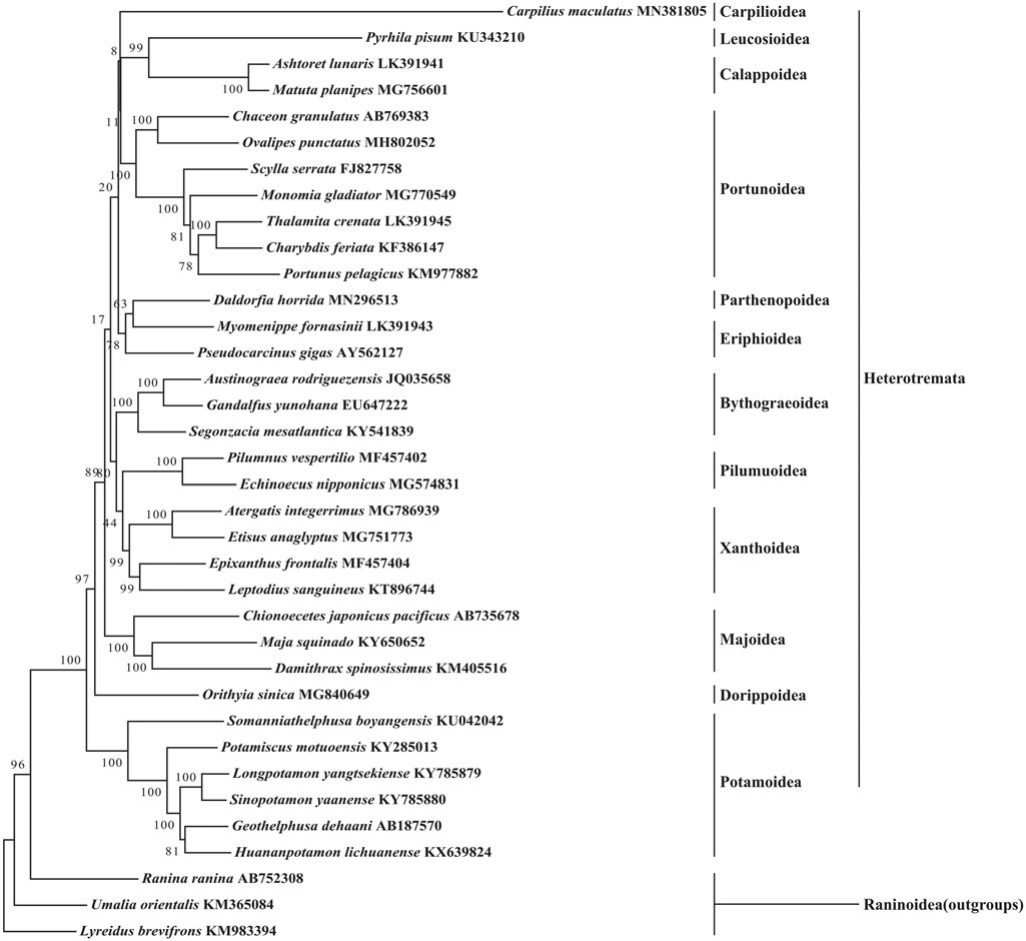
The maximum likelihood tree of *C. maculatus* and 35 other species in Heterotremata based on 13 PCGs. *Lyreidus brevifrons, Umalia orientalis* and *Ranina ranina* were used as outgroups.
